# Ultra-High-Performance Liquid Chromatography–Tandem Mass Spectrometry Analysis of Δ9-Tetrahydrocannabinol and Cannabidiol in Commercial Suk-Saiyasna Herbal Remedy: Applying Hansen Solubility Parameters for Sample Extraction to Ensure Regulatory Compliance

**DOI:** 10.3390/ph17111502

**Published:** 2024-11-08

**Authors:** Suwimon Sumontri, Wanna Eiamart, Sarin Tadtong, Weerasak Samee

**Affiliations:** 1 Department of Pharmaceutical Chemistry, Faculty of Pharmacy, Srinakharinwirot University, Nakhon Nayok 26120, Thailand; suwimon.puk@g.swu.ac.th; 2 Technical and Planning Division, Department of Thai Traditional and Alternative Medicine, Ministry of Public Health, Nonthaburi 11000, Thailand; 3 Chula Pharmacokinetic Research Center, Faculty of Medicine, Chulalongkorn University, Bangkok 10330, Thailand; wanna.eiamart@g.swu.ac.th; 4 Department of Pharmacognosy, Faculty of Pharmacy, Srinakharinwirot University, Nakhon Nayok 26120, Thailand; sarin@g.swu.ac.th

**Keywords:** cannabis, tetrahydrocannabinol, cannabidiol, extraction, chromatography, narcotic, regulation

## Abstract

Background: Suk-Saiyasna is a traditional Thai herbal remedy that comprises 12 distinct herbs. Among these, cannabis leaves constitute 12 of the total 78 components in this formulation. This study specifically examines the portion of the cannabis plant, which accounts for approximately 15.8% of the overall herbal composition used in the entire remedy. According to the Thailand Narcotics Act of 2022, the Δ9-tetrahydrocannabinol (Δ9-THC) concentration in herbal extracts must not exceed 0.2% by weight. This study aims to quantify the levels of Δ9-THC and cannabidiol (CBD) in commercial Suk-Saiyasna products. Methodology: This research utilizes Hansen Solubility Parameters (HSPs) to identify the optimal solvent for ultrasonic extraction, thereby maximizing cannabinoid yield. An advanced method was developed employing ultra-high-performance liquid chromatography–tandem mass spectrometry (UHPLC-MS/MS), compliant with AOAC standards to meet regulatory guidelines. The method validation emphasized specificity, linearity, sensitivity, accuracy, and precision. Results: Dichloromethane was chosen due to its favorable HSP values, enabling highly efficient extraction of Δ9-THC and CBD, achieving recovery rates of over 99.9% after the second extraction. This investigation benefits from the accuracy of the UHPLC-MS/MS technique in quantifying cannabinoids in commercial products, with Δ9-THC concentrations observed between 0.00231% and 0.14218%, and CBD concentrations ranging from 0.00002% to 0.01541%, all remaining below the legal limit. Conclusions: The variability in cannabinoid concentrations among various commercial products highlights the need for standardization in the herbal industry. This finding underscores the critical role of rigorous quality control measures in ensuring the safety and efficacy of cannabis-derived products.

1. Introduction

Suk-Saiyasna is a traditional Thai medicine recipe containing 12 herbal ingredients in specific proportions: camphor one part; *Azadirachta indica* A. Juss. leaves—two parts; *Kleinhovia hospita* L. root—three parts; *Cinnamomum bejolghota* (Buch.-Ham.) Sweet bark—four parts; *Nigella sativa* L. seeds—five parts; *Aucklandia lappa* (Decne.) Decne. root—six parts; *Myristica fragrans* Houtt. fruit—seven parts; *Mesua ferrea* L. flowers—eight parts; *Piper nigrum* L. fruit—nine parts; *Zingiber officinale* Roscoe rhizome—ten parts; *Piper retrofractum* Vahl. fruit—eleven parts; and *Cannabis sativa* L. leaves—twelve parts (12 out of a total of 78 parts, equivalent to 15.38%). It is used as a multipurpose remedy, including as a sedative [1]. Recent studies suggest that a related herbal remedy may enhance the effects of γ-aminobutyric acid type A (GABAA) receptor-targeting drugs, potentially influencing sleep and sedation [2]. The growing interest in cannabinoids like Δ9-tetrahydrocannabinol (Δ9-THC) and cannabidiol (CBD) for therapeutic use has led to challenges in ensuring safety, quality, and regulatory compliance of cannabis-derived products, particularly in accurately measuring Δ9-THC and CBD concentrations across different formulations [3,4]. In accordance with the Thailand Narcotics Act 2022, which came into effect on 9 June 2022, specific components of the cannabis plant have been reclassified and are no longer considered Category 5 narcotics. These components comprise the bark, stems, fibers, branches, roots, and leaves devoid of flowers or buds. Moreover, any extracts containing Δ9-THC are required to have a concentration not exceeding 0.2% by weight [5].

The extraction of Δ9-THC from cannabis involves various methods, each with its own advantages and limitations. The main extraction techniques discussed are the following: (1) Solvent extraction: Uses organic solvents like ethanol, butane, or hexane. Ethanol is noted for its efficiency and safety [6]. (2) Supercritical fluid extraction (SFE): Primarily uses supercritical CO_2_, allowing selective extraction of cannabinoids while minimizing undesirable compounds [7,8,9]. (3) Soxhlet extraction: A traditional method known for thoroughness but criticized for long extraction times and high solvent use [10]. (4) Microwave-assisted extraction (MAE): Uses microwave energy to enhance extraction, reducing time and increasing yields [11]. (5) Pressurized hot water extraction (PHWE): An environmentally friendly method using high-temperature, high-pressure water [12]. Each method affects the yield, purity, extraction time, and solvent usage differently. The choice of method depends on the specific requirements of the extraction process and the intended use of the final product.

The Hansen Solubility Parameter (HSP) framework offers a sophisticated approach to understanding compound solubility, particularly in herbal extraction contexts. Developed by Charles Hansen, HSPs decompose a substance’s cohesive energy density into three components: dispersion force (δd), polar force (δp), and hydrogen bonding (δh) parameters [13,14]. This tripartite division enables a nuanced comprehension of solvent–phytochemical interactions, crucial for optimizing extraction processes. The total Hansen Solubility Parameter (δt) is calculated as δt = √(δd^2^ + δp^2^ + δh^2^). This calculation facilitates the assessment of solvent efficacy in extracting specific compounds from plant materials [15]. Solvents that possess HSP values close to those of the target compounds generally result in higher extraction efficiencies, thereby reducing the reliance on empirical trial-and-error methods [16]. In herbal extraction, HSPs guide solvent selection to maximize desired phytochemical recovery while minimizing unwanted substance extraction. This approach aligns with green chemistry principles, promoting environmentally friendly solvents and reducing waste [17]. The Hansen Solubility Parameters provide a valuable tool for optimizing herbal extraction processes. By facilitating the selection of appropriate solvents based on their interaction with target phytochemicals, HSPs can enhance extraction efficiency, reduce environmental impact, and improve the overall quality of herbal products.

Group contribution methods (GCMs) and Hansen Solubility Parameters (HSPs) are essential for understanding solubility phenomena in various materials. GCMs are computational techniques used to estimate the HSPs of compounds based on their molecular structures. The fundamental principle of GCMs involves the decomposition of a molecule into its constituent functional groups, each of which is assigned a specific contribution to the overall property of interest. This methodology allows for the estimation of interaction parameters and thermodynamic properties using a limited set of empirical data, which is particularly advantageous in the context of complex mixtures and reactions [18,19].

In 1987, the United Nations Office on Drugs and Crime (UNODC) published the first manual on recommended methods for testing cannabis (ST/NAR/8), which was revised in 2009 and most recently updated in 2022. The UNODC recommended various analytical techniques and methodologies for the identification and analysis of cannabinoids in cannabis products. These techniques include simple color tests, thin-layer chromatography (TLC), gas chromatography–flame ionization detection (GC-FID), gas chromatography–mass spectrometry (GC-MS), liquid chromatography (LC), and liquid chromatography–mass spectrometry (LC-MS or LC-MS/MS), as well as combinations of these methods [20]. Furthermore, a novel methodology utilizing high-performance liquid chromatography with ultraviolet detection (HPLC-UV) has been developed, demonstrating success in analyzing cannabinoids within cannabis products [21]. 

Liquid chromatography–tandem mass spectrometry (LC-MS/MS) has become a crucial analytical tool in herbal medicine due to its ability to simultaneously identify and quantify multiple components in complex herbal samples [22,23]. Recent studies have demonstrated its effectiveness in analyzing traditional herbal formulas, detecting adulterants, and ensuring quality control [24]. LC-MS/MS can analyze complex herbal matrices, integrate with other analytical techniques, and facilitate rapid identification of compounds through automated dereplication processes [25]. As the herbal product market grows, LC-MS/MS plays an indispensable role in ensuring the safety, efficacy, and quality of these products. The technique’s versatility and comprehensive analytical capabilities make it essential for regulatory compliance and consumer safety in the field of herbal medicine.

In this study, we conducted a comprehensive quantitative analysis of Δ9-THC and CBD in commercial herbal remedies using UHPLC-MS/MS to address the separation issues associated with HPLC-UV. Unlike traditional extraction methods, which often involve the use of organic solvents with varying degrees of efficiency and reproducibility, we propose a novel approach based on Hansen Solubility Parameters (HSPs) for sample extraction. HSPs offer a systematic framework for predicting the solubility behavior of compounds in different solvents, thereby enabling the selection of optimal extraction conditions for cannabinoids with enhanced accuracy and reproducibility. Our methodology not only facilitates the efficient extraction of Δ9-THC and CBD from complex herbal matrices but also addresses critical concerns related to regulatory compliance. By employing HSP-based extraction, we aim to ensure consistency in cannabinoid quantification across different samples. These methodologies not only enhance extraction efficiency and yield but also promote the standardization of protocols, which is essential for obtaining reliable and reproducible results in cannabinoid research and its applications.

## 2. Results and Discussion

### 2.1. Solvent Selection

#### 2.1.1. Solvent Selection by Hansen Solubility Parameters

Hansen Solubility Parameters (HSPs) provide valuable insights into the solubility behavior of compounds by breaking down solubility into three components: dispersion forces (δd), polar forces (δp), and hydrogen bonding forces (δh). In this study, we employed the group contribution method to calculate the HSPs for Δ9-THC and CBD based on their molecular structures. Δ9-THC and CBD are two major cannabinoids found in the *Cannabis sativa* plant. Their molecular structures consist of a combination of aromatic rings, aliphatic chains, and hydroxyl functional groups. These structural features influence the intermolecular interactions and, consequently, the solubility behavior of these compounds. The group contribution method involves breaking down the molecular structure of a compound into functional groups and assigning HSP values to each group based on empirical data. These group contributions are then summed up to obtain the overall HSP values for the compound, as shown in Table 1 and Table 2. Based on the group contribution method, the calculated HSP values for Δ9-THC and CBD are as follows:

Δ9-THC: Dispersion force (δd) = 19.0777 MPa1/2; 

polar force (δp) = 3.5213 MPa1/2

Hydrogen bonding force (δh) = 6.9027 MPa1/2.

CBD: Dispersion force (δd) = 18.5696 MPa1/2

Polar force (δp) = 2.5886 MPa1/2; 

hydrogen bonding force (δh) = 13.1202 MPa1/2.


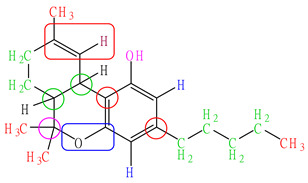


**Table 1:** Prediction of Hansen Solubility Parameters of Δ9-THC.

Group	Ni	Ci			(NiCi) p	(NiCi) d	(NiCi) hb
First Order		δd	δp	δh			
-CH_3_	4	−0.9714	−1.6448	−0.7813	−3.8856	−6.5792	−3.1252
-CH_2_	6	−0.0269	−0.3045	−0.4117	−0.1614	−1.8270	−2.4702
AC	2	0.8496	0.6187	0.0084	1.6992	1.2374	0.0168
ACH	2	0.1105	−0.5303	−0.4305	0.2210	−1.0606	−0.8610
ACOH	1	0.5288	1.101	6.958	0.5288	1.1010	6.9580
>C<	1	1.2686	2.0838	0.0866	1.2686	2.0838	0.0866
-CH<	2	0.645	0.6491	−0.2018	1.2900	1.2982	−0.4036
-CH=C<	1	0.5372	−0.9024	−1.8872	0.5372	−0.9024	−1.8872
**Second order**	**Mj**						
AC-O-C	1	0.2568	0.8153	0.6092	0.2568	0.8153	0.6092
ΣNiCi + ΣMjCi					1.7546	−3.8335	−1.0766
Constant I					17.3231	7.3548	7.9793
ΣNiCi + ΣMjCi + C					19.0777	3.5213	6.9027


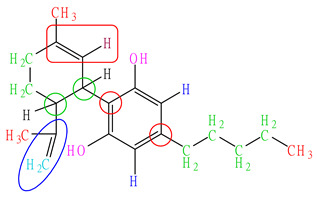


**Table 2:** Prediction of Hansen Solubility Parameters of CBD.

Group	Ni	Ci			(NiCi) p	(NiCi) d	(NiCi) hb
First Order		δd	δp	δh			
-CH_3_	3	−0.9714	−1.6448	−0.7813	−3.8856	−6.5792	−3.1252
-CH_2_	6	−0.0269	−0.3045	−0.4117	−0.1614	−1.8270	−2.4702
AC	2	0.8496	0.6187	0.0084	1.6992	1.2374	0.0168
ACH	2	0.1105	−0.5303	−0.4305	0.2210	−1.0606	−0.8610
ACOH	2	0.5288	1.101	6.958	1.0576	2.2020	13.9160
CH_2_=C<	1	−0.4829	−0.7794	−0.826	−0.4829	−0.7794	−0.8260
-CH<	2	0.645	0.6491	−0.2018	1.2900	1.2982	−0.4036
-CH=C<	1	0.5372	−0.9024	−1.8872	0.5372	−0.9024	−1.8872
ΣNiCi + ΣMjCi					1.2465	−4.7662	5.1409
Constant I					17.3231	7.3548	7.9793
ΣNiCi + ΣMjCi + C					18.5696	2.5886	13.1202

The calculated HSPs for Δ9-tetrahydrocannabinol (Δ9-THC) and cannabidiol (CBD) provide critical insights into their solubility characteristics in various solvent systems. The HSP framework, developed by Charles Hansen, divides the total solubility parameter into three components: dispersion forces (δD), polar forces (δP), and hydrogen bonding forces (δH) [26,27]. Dispersion forces indicate a compound’s ability to interact with non-polar solvents, while polar forces reflect interactions with polar solvents. Hydrogen bonding forces describe the capacity of a compound to form hydrogen bonds with solvent molecules [28]. 

Solvents with HSP values closely matching those of Δ9-THC and CBD were selected for extraction. The compatibility between a solvent and the solute of interest can be quantified by the radius interaction (Ra), calculated using the following equation:

$$R_{a}^{2}=4\;{\left(\delta_{di}-\delta_{dj}\right)}^{2}+{\left(\delta_{pi}-\delta_{pj}\right)}^{2}+{\left(\delta_{hi}-\delta_{hj}\right)}^{2}$$

where i is the HSPs of the solute, and j is the HSPs of a solvent.

The radius of interaction (Ra) is a concept derived from Hansen Solubility Parameters (HSPs) and serves to define a ‘solubility sphere’. Within this sphere, effective solvents are located, while ineffective solvents are positioned outside of it. The center of the sphere corresponds to the HSPs of the solute, and the radius is determined by the calculated HSP values and the concept of relative energy difference (RED). A smaller Ra distance signifies a more suitable solvent for solute extraction. By comparing the HSP values of Δ9-THC and CBD, predictions regarding their solubility behavior in different solvent systems can be made. Solvents with HSP values that closely match those of the cannabinoids are expected to exhibit superior solubility characteristics. For instance, as presented in Table 3, the HSP values of dichloromethane are notably close to those of Δ9-THC (Ra = 3.38) and CBD (Ra = 7.98), suggesting that dichloromethane is an effective solvent for extracting these cannabinoids from herbal materials [29]. The selection of dichloromethane for the complete extraction of Δ9-THC provides the accuracy and precision required for regulatory compliance in the analysis of commercial herbal remedies.

For green solvents with interaction radius (Ra) values close to dichloromethane, such as ethyl acetate (Ra = 6.80 for Δ9-THC and Ra = 8.55 for CBD), and commonly used green solvents for extraction like ethanol (Ra = 15.07 for THC and Ra = 10.43 for CBD), further studies on extraction efficiency may be necessary through practical experiments to determine whether they can effectively serve as substitutes for dichloromethane in the extraction of CBD and Δ9-THC in regulatory contexts.

#### 2.1.2. Effect of Dichloromethane, Ethyl Acetate, and Ethanol on Extraction of Δ9-THC and CBD from Cannabis Powder

**Figure 1:**
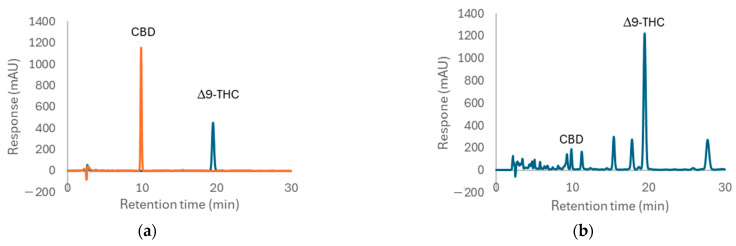

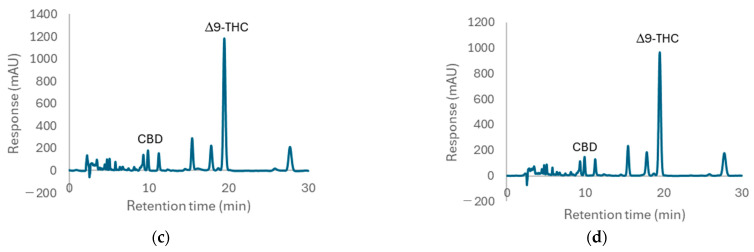
Chromatograms of (**a**) overlaid standard CBD (orange) and Δ9-THC (blue), (**b**) dichloromethane extract, (**c**) ethanol extract, and (**d**) ethyl acetate extract, detected at 208 nm UV.

Dichloromethane, in conjunction with green solvents such as ethyl acetate and ethanol, was selected as the solvent for the extraction of Δ9-THC and CBD from the combined leaves and inflorescences of the cannabis sample. This selection aimed to optimize the yield of CBD in the extract. A mixture of solvent and herbal powder was prepared at a ratio of 10:1, subjected to centrifugation, and subsequently extracted using ultrasonic extraction for 30 min, with temperature control maintained at or below 30 degrees Celsius. The extraction process was repeated three times consecutively, and the combined extracts were analyzed using HPLC-PDA, which has been modified from the method established by Analakkattillam et al. [21]. The chromatograms for standard CBD (retention time = 9.89 min) and standard Δ9-THC (retention time = 19.53 min), detected at 208 nm, are illustrated in Figure 1a,b. The chromatograms for the extracted samples are presented in Figure 1c,d. The peak areas of Δ9-THC and CBD extracted using different solvents are summarized in Table 4. The dichloromethane extract exhibited the highest peak area (peak area CBD = 2185 and Δ9-THC = 25,793), demonstrating its effectiveness as determined by HSPs. In contrast, the extracts obtained using ethyl acetate (peak area CBD = 1628 and Δ9-THC = 19,262) and ethanol (peak area CBD = 1959 and Δ9-THC = 23,037) exhibited lower concentrations compared to those extracted with dichloromethane.

These findings suggest that, while ethanol is a green solvent suitable for extracting samples intended for commercial and human use, dichloromethane remains the preferred solvent for regulatory approaches that require complete extraction and accurate analysis of CBD and Δ9-THC.

#### 2.1.3. Stability of CBD and Δ9-THC Under Ultrasonic Extraction Conditions

To evaluate whether this extraction method leads to the degradation of CBD and Δ9-THC, we assessed the stability of standard ethanolic solutions of CBD and Δ9-THC at a concentration of 100 µg/mL under the specified conditions. The results presented in Table 5 indicate that the remaining percentage of the CBD standard was 98.75%, while the remaining percentage of the Δ9-THC standard was 100.11%.

**Figure 2:**
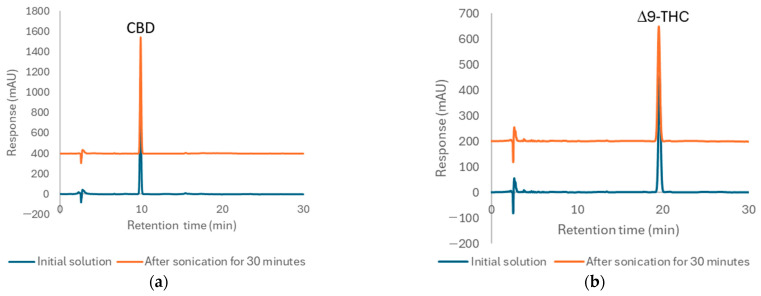
Chromatograms of the initial standard solution and the solution after sonication for 30 min for (**a**) CBD and (**b**) Δ9-THC.

The chromatograms of standard CBD and Δ9-THC following sonication closely resembled the initial standard solutions, with no degradation peak observed, as shown in Figure 2. These findings suggest that both standards maintained good stability under the extraction conditions. Consequently, this extraction method is considered suitable for isolating CBD and Δ9-THC from sample matrices for regulatory applications, as it facilitates a relatively rapid extraction process without compromising the integrity of the target analytes.

### 2.2. Preparation of Suk-Saiyasna Crude Extracts

The extraction study aimed to prepare Suk-Saiyasna crude extracts from one in-house prepared herbal powder sample and six commercially available brand samples. Each 30 g sample was subjected to extraction using 300 mL of dichloromethane as the solvent. The extraction process involved sonication of the herbal powder for 30 min, repeated three times to maximize yield. Table 6 presents the results, showing that crude extract yields (% yield) varied significantly, ranging from 3.12% to 33.89%. Notably, three commercial brand samples identified as A, B, and D had percentage yields comparable to the in-house prepared sample, which yielded 16.38%. In contrast, Brand C displayed a significantly higher yield of 33.89%, nearly double that of the in-house preparation, suggesting superior extraction efficiency or higher concentrations of active compounds in its formulation. Conversely, Brands E and F yielded less than 6%, indicating substantially lower extraction efficiency. These findings highlight the impact of variations in raw materials and manufacturing processes on the extraction efficiency of herbal formulations. Previous studies have noted that differences in plant material quality, including factors such as plant age, harvesting time, and processing methods, can lead to substantial discrepancies in extraction yields [28,30]. Furthermore, the choice of solvent and extraction technique is critical in determining phytochemical extraction efficiency [31]. Dichloromethane, known for its ability to dissolve a wide range of non-polar compounds, may have contributed to the variations observed in yields across the different brands [32]. This study underscores the importance of standardizing extraction methods and raw material sourcing in the herbal industry to ensure consistent product quality. Variability in extraction yields can affect the efficacy and safety of herbal products, particularly in therapeutic applications [33]. Future research should focus on optimizing extraction parameters and investigating the phytochemical profiles of different samples to better understand the factors influencing yield and efficacy.

### 2.3. Analysis of Δ9-THC and CBD in Suk-Saiyasna Herbal Remedy Extracts by HPLC-PDA

**Figure 3:**
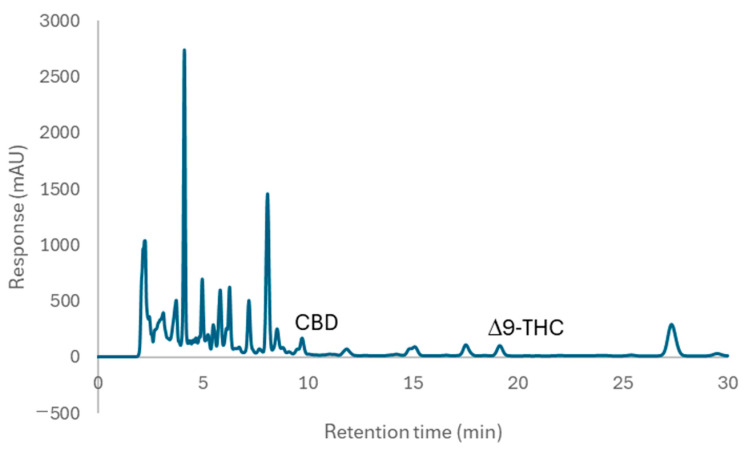
Chromatogram of the extract from the Suk-Saiyasna herbal remedy in commercial brand A, detected at 208 nm UV.

The HPLC-UV method for the quantification of Δ9-THC and CBD in the Suk-Saiyasna herbal remedy has been developed through modifications of the method established by Analakkattillam et al. [21]. This modified approach employs an octadecylsilane column (250 mm × 4.6 mm, 5 μm) and utilizes a mobile phase composed of acetonitrile and water in a 75:25 ratio. The method operates at a flow rate of 1 mL/min, with an injection volume of 10 µL, and detection occurs at a wavelength of 208 nm, demonstrating success in analyzing Δ9-THC and CBD within cannabis extracts, as well as the stability of the standard mixture, as shown in Figure 1 and Figure 2. However, when the HPLC-UV method was applied to analyze the Suk-Saiyasna remedy, which has a complex composition, it was found that the method did not adequately enhance the resolution of CBD relative to other components, as illustrated in Figure 3. Consequently, UHPLC-MS/MS presents a viable alternative for the quantitative analysis of Δ9-THC and CBD, particularly when accuracy and reliability are required for regulatory purposes.

### 2.4. LC MS/MS Optimization

**Figure 4:**
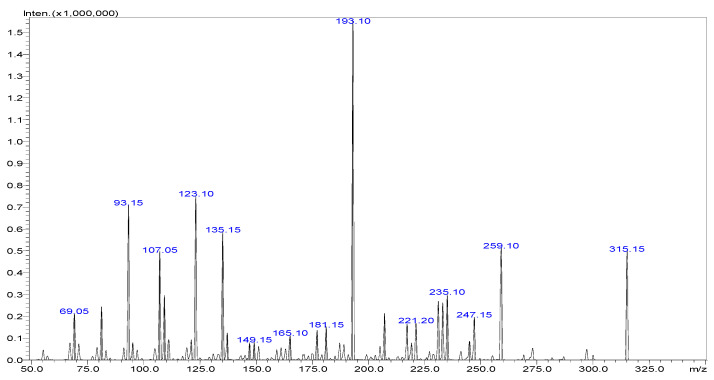
Mass fragmentation spectra of Δ9-THC in ESI positive mode.

**Figure 5:**
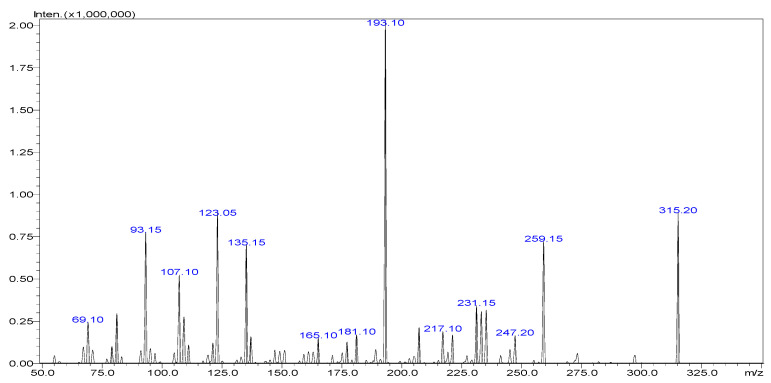
Mass fragmentation spectra of CBD in ESI positive mode.

**Figure 6:**
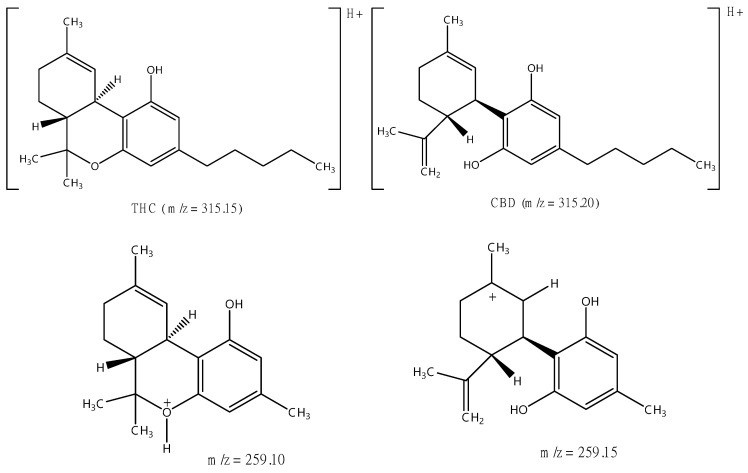

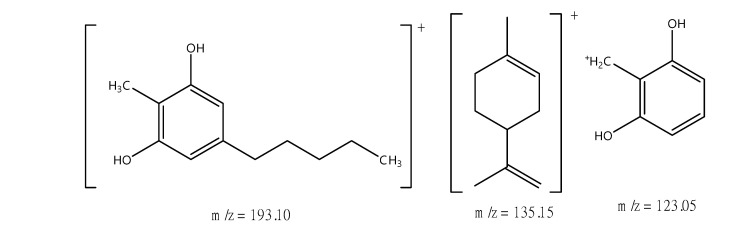
Proposed fragments of Δ9-THC and CBD in ESI positive mode.

The precursor ions selected for Δ9-THC and CBD typically correspond to the protonated molecular ions ([M + H]^+^) generated in the electrospray ionization (ESI) source. Fragmentation of these precursor ions in the collision cell of the mass spectrometer results in the generation of specific product ions characteristic of each analyte. Δ9-THC and CBD have a molecular weight of 314.45 g mol^−1^ and show a similar precursor to product ion transitions (315 > 123, 193, and 259), as shown in Figure 4, Figure 5 and Figure 6. Multiple reaction monitoring (MRM) transitions were optimized for the quantification of Δ9-THC and CBD.

The selection of appropriate MRM transitions is crucial for achieving high selectivity and sensitivity in the analysis of Δ9-THC and CBD. The chosen transitions should exhibit minimal interference from background noise and matrix components while maximizing the signal-to-noise ratio for the target analytes. The selected MRM transitions for the analysis of Δ9-THC and CBD, along with their corresponding precursor and product ions, are summarized in Table 7. It was established that the quantification ion, which yields a higher signal, corresponds to the transition 315 > 193 for both Δ9-THC and CBD. Consequently, it is imperative that the analytes be separated chromatographically; otherwise, accurate quantification is not feasible.

By monitoring these specific MRM transitions, the UHPLC-MS/MS system can selectively detect and quantify Δ9-THC and CBD in complex sample matrices with high sensitivity and accuracy. 

### 2.5. Method Validation

The validation of the analytical method for cannabinoid quantification was conducted in strict adherence to the guidelines established by the Association of Official Analytical Chemists (AOAC). This rigorous approach ensures the reliability and accuracy of the analytical procedure employed in this study [34,35,36,37,38].

#### 2.5.1. Specificity

**Figure 7:**
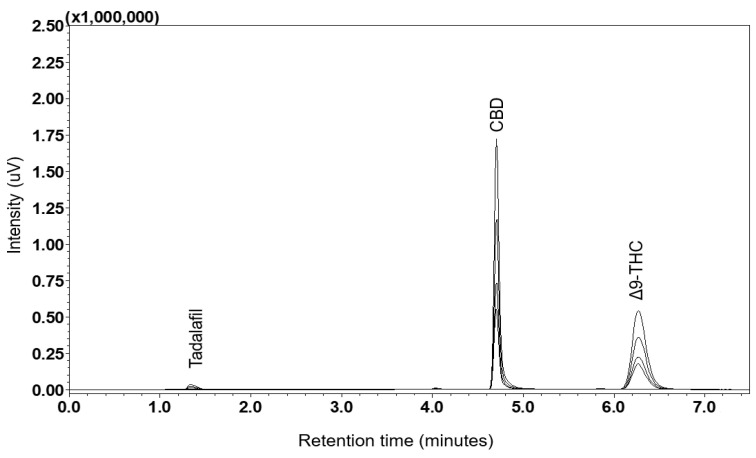
Comparison of overlaid chromatograms: Suk-Saiyasna formulation extract versus spiked standard solutions of CBD and Δ9-THC.

As illustrated in Figure 7, the chromatographic profile was obtained from the Suk-Saiyasna formulation extract after being spiked with standard solutions of CBD and Δ9-THC. The chromatogram demonstrates the separation and detection of these cannabinoids within the complex matrix of the herbal formulation. The retention times were observed to be 4.7 and 6.2 min for CBD and Δ9-THC, respectively. The overlaid chromatograms of the standards and the sample exhibited identical retention times and perfectly overlapped. Notably, no interference was detected from other components present in the sample formulation, thus demonstrating the specificity of the analytical method employed.

#### 2.5.2. Linearity and Range

As presented in Table 8, the analysis of CBD and Δ9-THC standard solutions in methanol solvent was conducted across a concentration range of 0.005–20 µg/mL. The average peak areas and corresponding concentrations were utilized to construct calibration curves. The results indicate that calibration curves 1, 2, and 3 from three different validation days exhibited coefficient of determination (r^2^) values of 0.9999, which translates to a correlation coefficient (r) of 0.9999. These values surpass the AOAC standard criteria, which stipulate a minimum r value of 0.995. This high degree of linearity demonstrates the robust performance and reliability of the analytical method within the specified concentration range.

#### 2.5.3. LOD and LOQ

Utilizing the linear equation data, the limit of detection (LOD) and limit of quantification (LOQ) were calculated employing the following formulas:

LOD = 3.3 × (Standard Deviation of Y-intercept)/(Mean of Slope)

LOQ = 10 × (Standard Deviation of Y-intercept)/(Mean of Slope)

As illustrated in Table 8, the calculated LOD and LOQ values for CBD were determined to be 0.3973 ng/mL and 1.2039 ng/mL, respectively. Similarly, for Δ^9^-THC, the LOD and LOQ values were ascertained as 0.9706 ng/mL and 2.9412 ng/mL, respectively. These results demonstrate the method’s sensitivity and its capability to detect and quantify both cannabinoids at low concentrations in the sample matrix.

#### 2.5.4. Accuracy and Precision

The analysis of CBD and Δ9-THC employed a mass spectrometer as the detector, which demonstrates high specificity for both cannabinoids without interference from other components in the Suk-Saiyasna formulation. Accuracy and precision tests were conducted using CBD and Δ9-THC standard solutions in methanol solvent at concentrations of 10, 100, and 1000 ng/mL, with triplicate preparations for each concentration. As presented in Table 9, Δ9-THC exhibited average recovery rates ranging from 98.98% to 103.72%. The relative standard deviation (%RSD) for intra-day precision ranged from 0.59% to 2.09%, while the %RSD for inter-day precision was between 1.09% and 2.06%. For CBD, average recovery rates ranged from 97.78% to 100.15%, with intra-day precision %RSD values of 1.51% to 1.92% and inter-day precision %RSD values between 1.52% and 1.67%.

The Narcotics Act 2022, effective 9 June 2022, reclassified certain parts of the cannabis plant, excluding them from Category 5 narcotics [5]. These parts include bark, stem, fiber, branches, roots, and leaves without attached shoots or inflorescences. The act stipulates that extracts containing Δ9-THC must not exceed 0.2% by weight, with the same threshold applied to residues or remnants from cannabis extraction. In this study, CBD content was evaluated using the same 0.2% by weight threshold as Δ9-THC. When compared to the AOAC standards, which require a recovery rate between 90 and 108% and a %RSD not exceeding 3%, the analytical methods for both CBD and Δ9-THC met these criteria. These results confirm the reliability and accuracy of the developed analytical methods for both cannabinoids in the Suk-Saiyasna formulation.

The comprehensive validation of the analytical method for cannabinoid quantification in the Suk-Saiyasna formulation has confirmed its reliability, accuracy, and suitability for its intended purpose. This robust analytical approach provides a foundation for the quality control and regulatory compliance of this traditional herbal preparation.

### 2.6. The Extraction Efficiency of Dichloromethane in Extracting Δ9-THC and CBD from the Suk-Saiyasna Remedy

Extraction efficiency in cannabinoid formulations is of paramount importance, particularly when evaluating the completeness of cannabinoid extraction from raw materials. A prevalent challenge lies in the verification that all cannabinoids have been successfully extracted, especially in instances where the initial concentrations of cannabinoids within the sample are unknown. To ensure that all cannabinoids have been extracted from cannabis remedies, it is essential to quantify cannabinoid content across multiple extraction processes and to utilize standardized extraction methods that maximize yield. High-sensitivity analytical techniques, such as liquid chromatography–tandem mass spectrometry (LC-MS/MS), can provide the requisite data to accurately evaluate extraction efficiency, thereby ensuring that the reported values reflect the true cannabinoid content present in the original sample. This study assessed the recovery rate of the extraction protocol by spiking CBD and Δ9-THC standards into an in-house formulation prior to extraction, with recovery rates ranging from 94.53% to 97.48% for CBD and from 94.86% to 98.08% for Δ9-THC during the first round of extraction and approaching 100% in the second round of extraction for both compounds.

**Figure 8:**
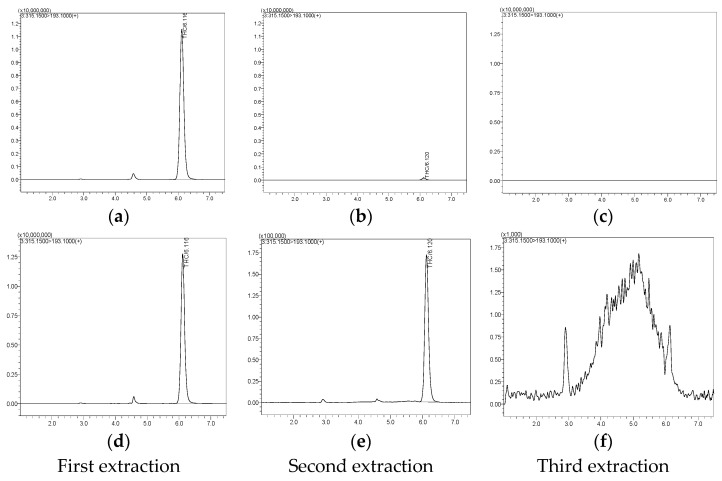
LC-MS/MS chromatograms of the first, second, and third extractions: (**a**–**c**) in-house Suk-Saiyasna remedy, (**d**–**f**) expanded chromatogram of the in-house Suk-Saiyasna remedy.

For systematic evaluation, the extraction efficiency of dichloromethane in isolating Δ9-THC and CBD from the matrix of the Suk-Saiyasna remedy was assessed. The extracts obtained from each extraction round were analyzed using UHPLC/MS/MS to quantify the levels of Δ9-THC and CBD. The extraction efficiency was determined by dividing the quantity of each cannabinoid obtained from individual extraction rounds by the total quantity obtained after three extraction rounds, providing a clear measure of solute transfer efficiency from the herbal matrix to the solvent phase. The results demonstrated an impressive extraction efficiency of 99.9% for both Δ9-THC and CBD after triplicate extractions with dichloromethane. As shown in Figure 8, the peaks for CBD and Δ9-THC are not visible by the third round of extraction until the signal is amplified by an additional factor of 1000, as demonstrated in Figure 8f, which permits the visualization of these peaks. This observation indicates that the three consecutive extraction processes successfully extracted nearly all the CBD and Δ9-THC present in the sample. Dichloromethane facilitated the extraction of over 99.9% of Δ9-THC and CBD by the second round, with nearly complete extraction achieved by the third round (assuming that complete extraction is attainable through three consecutive extraction processes) (Table 10). These findings highlight dichloromethane’s effectiveness as a solvent for cannabinoid extraction, confirming its suitability based on HSPs [29,30]. The high extraction efficiency observed in this study aligns with previous research emphasizing the importance of solvent polarity and extraction techniques in maximizing the yield of bioactive compounds from plant materials [31,32]. As a non-polar solvent, dichloromethane is particularly effective for extracting lipophilic compounds such as cannabinoids, which exhibit low solubility in polar solvents [33]. Dichloromethane’s ability to efficiently dissolve these compounds is further supported by its favorable HSP values, closely matching those of Δ9-THC and CBD [26]. Moreover, the results underscore the importance of employing multiple extraction rounds to achieve optimal yields. The diminishing returns observed in subsequent extractions suggest that while the majority of cannabinoids are extracted in the initial rounds, additional extractions may still yield valuable quantities, supporting the use of a triplicate extraction approach [27].

This method not only enhances overall extraction yield but also ensures more comprehensive recovery of the active constituents in the Suk-Saiyasna remedy. These findings affirm the efficacy of dichloromethane as a solvent for extracting Δ9-THC and CBD from the Suk-Saiyasna remedy. The high extraction efficiencies achieved through multiple extraction rounds demonstrate the solvent’s capability to effectively transfer these cannabinoids from the herbal matrix, providing a robust basis for its application in cannabinoid extraction processes.

### 2.7. Determination of Δ9-THC and CBD Contents in Suk-Saiyana Herbal Remedy

The Δ9-THC and CBD content in the Suk-Saiyasna herbal remedy involved extracting crude samples from one in-house prepared herbal powder and six commercially available brand samples. Each sample was derived from 30 g of powder, which included 4.61 g (15.38%) of cannabis leaves. The extracts were prepared at a concentration of 10 mg/mL to quantify the levels of Δ9-THC and CBD using UHPLC-MS/MS employing MRM. Tadalafil was utilized as an internal standard (IS), added prior to injection to minimize volumetric error. The area ratio between the target compounds and the IS was employed to calculate the sample concentration. Compound verification was carried out using the advanced LCMS-8060 detector, which provides both qualifier and quantifier capabilities, offering high selectivity and sensitivity.

The results of the accuracy and precision testing of the quality control (QC) samples at low, medium, and high concentrations are presented in Table 9, indicating that the percentage recovery values fall within the acceptable range. These findings further validate that the analytical method is not affected by matrix effects from non-target compounds in the samples or the internal standard. Consequently, we confirm that a 0.1 µL injection volume yields accurate analytical results. However, to enhance the accuracy and precision of the analytical measurements, larger injection volumes may be employed.

Establishing an intensity ratio of 30% between the quantifier (target ion) and qualifier (reference ion) ions (see Figure 7) enhances the accuracy of Δ9-THC and CBD measurements, including the internal standard. This approach ensures that the results are specific to the intended substances and are free from contamination by other compounds, corroborating the assertion that the method is unaffected by matrix effects.

**Figure 9:**
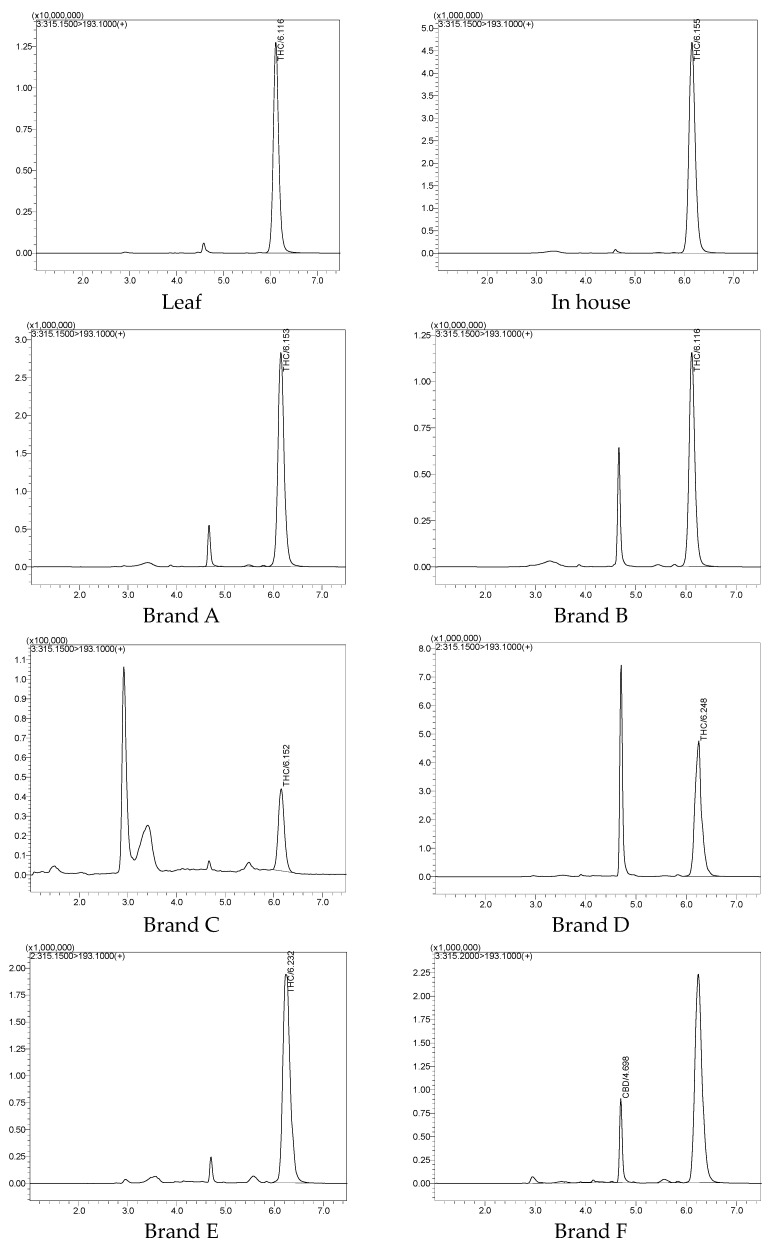
LC-MS/MS chromatograms of leaf, in-house, and commercial crude extracts.

As illustrated in Table 11 and Table 12 and Figure 9, the CBD and Δ9-THC contents in the cannabis leaves and the in-house formulation are comparable. This similarity supports the assertion that the extraction and analysis methods are effective for both single herbs and complex herbal mixtures. In contrast, the CBD content in the cannabis leaves from different commercial brands varied significantly, ranging from 0.00002% to 0.01541%. Similarly, the Δ9-THC concentrations in the crude extracts were analyzed, revealing values between 0.00231% and 0.14218%, all of which are within the legal limit of 0.2% [5]. This finding aligns with the existing literature, emphasizing the need to monitor cannabinoid levels to ensure compliance with regulatory standards [39].

Considerable variation was observed in the chromatograms shown in Figure 9 and in the levels of active compounds among different brands, as illustrated in Table 11 and Table 12. Brands A, B, and E exhibited Δ9-THC concentrations comparable to the in-house standard of approximately 0.09%, with Brand A showing the highest concentration. Conversely, Brand C had the lowest Δ9-THC levels. In terms of CBD content, most brands exceeded the in-house standard of 0.00021%, except for Brand C, which again showed the lowest concentration. Brand B demonstrated the highest CBD content among the samples analyzed. The increasing prevalence of cannabis products in the commercial market has raised concerns regarding the balance of cannabinoids, particularly the ratio of CBD to Δ9 THC. This research explores the implications of higher CBD content in commercial products compared to in-house formulations, focusing on the potential for CBD to mitigate the side effects associated with Δ9-THC. These variations in cannabinoid concentrations highlight the influence of sourcing and manufacturing practices on the quality and efficacy of herbal remedies. Previous studies indicate that the phytochemical profiles of cannabis products can be significantly affected by factors such as cultivation methods, plant genetics, and extraction techniques [40,41]. 

The concentration of CBD in commercial cannabis products is highly variable, and this variability significantly influences the pharmacological effects observed in vivo. Numerous studies have established a clear relationship between CBD concentration and its therapeutic outcomes, underscoring the importance of standardized dosing in clinical applications. One of the primary areas where CBD concentration plays a critical role is in its interaction with other cannabinoids, particularly Δ9-THC. Cuttler et al. [42] found that the presence of CBD can influence the effects of Δ9-THC, with some evidence suggesting that CBD may modulate Δ9-THC’s effects, potentially leading to different therapeutic outcomes. Furthermore, Englund et al. [43] reported that CBD can mitigate the psychotropic effects of Δ9-THC, indicating that the balance of these cannabinoids is crucial for achieving desired therapeutic outcomes without adverse effects.

The preparation of an in-house sample of the Suk-Saiyasna remedy, as opposed to relying on commercially available products, is essential for ensuring quality control (QC) and quality assurance (QA) as well as consistency in therapeutic effects. Due to variations in extraction yields and cannabinoid concentrations among different commercial brands, developing a standardized in-house formulation is critical. This approach allows us to implement rigorous QC and QA measures, including the selection of high-quality raw materials, standardization of extraction methods, and comprehensive testing of the final product. Furthermore, it helps ensure compliance with regulatory standards, which increasingly emphasize product consistency and safety in the evolving cannabis industry. 

The observation of varying cannabinoid concentrations among products indicates that manufacturers may employ different cannabis strains or processing methodologies, resulting in inconsistencies in the composition of the final products [44]. Furthermore, these findings highlight the critical need for stringent QC measures in the production of herbal remedies. Significant variations in Δ9-THC and CBD concentrations across brands may impact both therapeutic efficacy and safety, particularly concerning cannabinoid-based therapies. By standardizing extraction methodologies, conducting regular testing, ensuring traceability, educating consumers, and enhancing regulatory oversight, the cannabis industry can enhance product consistency and safety.

These measures are essential for fostering consumer trust and ensuring that cannabis products provide the intended therapeutic benefits while maintaining safety. As the demand for cannabis-derived products continues to increase, maintaining consistent quality and precise labeling will be paramount for consumer safety and regulatory compliance [38].

## 3. Materials and Methods

### 3.1. Materials

Chemical reference standards of Δ9-THC and CBD, as well as cannabis leaves, were obtained from the Bureau of Drug Narcotics, Department of Medical Sciences, Ministry of Public Health, Nonthaburi, Thailand. Analytical-grade dichloromethane, ethanol, and formic acid, along with LC-MS-grade acetonitrile and methanol, were procured from Merck (Boston, MA, USA). Commercial Thai traditional herbal remedies known as ‘Suk-SaiYasna’, containing cannabis leaves comprising 15.38 percent of the overall ingredients, were obtained from various manufacturers in Thailand.

### 3.2. Methods 

#### 3.2.1. Solvent Selection for Solvent Extraction 

Hansen Solubility Parameters (HSPs) were calculated for Δ9-THC and CBD based on their molecular structures using the group contribution method. Dispersion force (*δd*), dipolar intermolecular force (*δp*), and the energy from hydrogen bonding (*δhb*) were determined using Equations (1)–(3), respectively.

(1) $$\delta_{d}=\left(\sum_{i}N_{i}C_{i}+W\sum_{j}M_{j}D_{j}+17.3231\right){\;MPa}^{1/2}$$

(2) $$\delta_{p}=\left(\sum_{i}N_{i}C_{i}+W\sum_{j}M_{j}D_{j}+7.3548\right)\;{MPa}^{1/2}$$

(3) $$\delta_{hb}=\left(\sum_{i}N_{i}C_{i}+W\sum_{j}M_{j}D_{j}+7.9793\right)\;{MPa}^{1/2}$$

where *Ci* represents the contribution of the first-order group of type *i* that appears *Ni* times in the target structure, and *Dj* is the contribution of the second-order group of type *j* that appears *Mj* times in the target structure. The constant *W* is equal to 0 for compounds without second-order groups and equal to 1 for compounds which have second-order group.

Solvents with HSP values closely matching those of Δ9-THC and CBD were selected for extraction. The compatibility between a solvent and the solute of interest can be quantified by the radius (*Ra*), calculated using Equation (4).

(4) $$R_{a}^{2}=4\;{\left(\delta_{di}-\delta_{dj}\right)}^{2}+{\left(\delta_{pi}-\delta_{pj}\right)}^{2}+{\left(\delta_{hi}-\delta_{hj}\right)}^{2}$$

where *i* is the HSPs of the solute, and *j* is the HSPs of a solvent.

#### 3.2.2. Preparation of the In-House Suk-Saiyasna Remedy

Preparation of the in-house Suk-Saiyasna remedy involved weighing an herbal powder blend composed of 12 herbal ingredients in specific proportions: camphor 1 part; *Azadirachta indica* A. Juss. leaves—2 parts; *Kleinhovia hospita* L. root—3 parts; *Cinnamomum bejolghota* (Buch.-Ham.) Sweet bark—4 parts; *Nigella sativa* L. seed—5 parts; *Aucklandia lappa* (Decne.) Decne. root—6 parts; *Myristica fragrans* Houtt. fruit—7 parts; *Mesua ferrea* L. flowers—8 parts; *Piper nigrum* L. fruit—9 parts; *Zingiber officinale* Roscoe rhizome—10 parts; *Piper retrofractum* Vahl. fruit—11 parts; and *Cannabis sativa* L. leaves—12 parts.

#### 3.2.3. Preparation of Suk-Saiyasna Crude Extracts

Dichloromethane was utilized as an extractive solvent, selected for its compatibility with the Hansen Solubility Parameters (HSPs) of cannabinoids. The samples underwent extraction via a liquid–liquid extraction method. Specifically, 30 g of each sample was mixed with the chosen solvent in a 1:10 (*w*/*v*) ratio and subjected to sonication for 30 min. After extraction, the mixture was filtered using a vacuum pump to separate the solvent mixture, which contained the cannabinoids, from the solid residue. The solid residue was re-extracted three additional times. Subsequently, the entire solution was evaporated to dryness using a rotary evaporator. The resulting dry weight was measured and stored in a refrigerator at −20 degrees Celsius for further analysis.

#### 3.2.4. HPLC-UV Analysis

The HPLC system (Agilent, Santa Clara, CA, USA) comprised the 1260 Infinity II quaternary pump, 1260 Infinity II autosampler, 1260 Infinity II multi-column thermostat, and 1260 Infinity II photo diode array detector. The separation process occurred within an ACE 5 C18-AR column (Aberdeen, Scotland) (4.6 × 250 mm, 5 µm) paired with a C18 guard column. The mobile phase, consisting of acetonitrile and water in a fixed ratio of 75:25, maintained a steady flow rate of 1 mL/min. Prior to use, the mobile phase underwent degassing and was freshly prepared for each analysis. The detection wavelength was consistently set at 208 nm, with an injection volume of 10 µL. All analyses were performed at an operating temperature of 25 °C. Under these specified chromatographic conditions, the average retention times for Δ9-THC and CBD were determined to be 9.9 and 19.5 min, respectively.

#### 3.2.5. UHPLC-MS/MS Analysis

Ultra-high-performance liquid chromatography–tandem mass spectrometry (UHLC-MS/MS) was conducted using the LCMS-8060NX (Shimazu, Kyoto, Japan) and Lab Solution software version 5.99 SP2. The HPLC system comprised an LC-4D XS pump, a CTO-40S column oven, an SIL-40C autosampler, a DGU-405 degasser unit, and an FCV-11AL valve unit, all manufactured by Shimadzu Corporation, Kyoto, Japan. A gradient elution was performed on a Phenomenex Luna C18 column (Torrance, CA, USA) (100 × 2 mm, 3 µm particle size) with a guard column. The mobile phase consisted of two different solutions: 0.01% formic acid in 20mM ammonium formate (solution A) and acetonitrile (solution B). All solutions were degassed and filtered through a 0.22 μm pore size filter. The gradient elution started with initial conditions of 60% eluent B, maintained for 1 min, followed by a linear gradient to 80% from 1.01 to 1.5 min, held for 2.5 min, followed by another linear gradient to 60% from 4.01 to 4.5 min, maintaining this proportion until 7.6 min at a flow rate of 0.4 mL/min. The column temperature was maintained at 40 °C, the autosampler temperature at 15 °C, and the injection volume was 0.1 μL. Tandem mass spectrometry detection was performed using an electrospray ionization (ESI) source in positive ion mode (ESI+) for the detection of Δ9-THC and CBD. Multiple reaction monitoring (MRM) transitions were optimized for the quantification of Δ9-THC and CBD. Tadalafil was used as an internal standard. The analytical method was validated for specificity, linearity, precision, accuracy, and robustness according to AOAC guidelines.

## 4. Conclusions

This study emphasizes the effectiveness of Hansen Solubility Parameters (HSPs) in enhancing the extraction of Δ9-THC and CBD from commercial herbal formulations. By employing dichloromethane, identified through HSP analysis, the extraction efficiency exceeded 99.9%, enabling precise quantification of cannabinoids. The analytical method, validated by UHPLC-MS/MS, aligns with AOAC standards and exhibits robust specificity, linearity, sensitivity, accuracy, and precision, affirming its appropriateness for regulatory compliance. An analysis of the Suk-Saiyasna remedy, a crude extract with 4.61 g (15.38%) of cannabis leaves, showed a CBD content ranging from 0.00002% to 0.01541%. Similarly, the Δ9-THC content in the crude extract was analyzed, revealing concentrations from 0.00231% to 0.14218%, all within the legal limit of 0.2%. These findings highlight the need for manufacturing standardization to ensure consistent quality and potency across commercial herbal remedies. Future research should explore the pharmacological effects of these cannabinoids in clinical settings to further investigate their therapeutic potential.

## Figures and Tables

**Table 3:** Calculated radius (Ra) of HSP values of Δ9-THC and CBD with common solvents.

Name	Hansen Solubility Parameters			Ra	
Solute	δd	δp	δh	Δ9-THC	CBD
Δ9-THC	19.08	3.52	6.90		
CBD	18.57	2.59	13.12		
**Solvent**					
Dichloromethane	18.2	6.3	6.1	3.38	7.98
Toluene	18	1.4	2	5.76	11.24
Ethyl Acetate	15.8	5.3	7.2	6.80	8.55
Cyclohexane	16.8	0	0.2	8.84	13.64
Diethyl Ether	14.5	2.9	4.6	9.46	11.79
Acetone	15.5	10.4	7	9.93	11.67
Butan-1-ol	16	5.7	15.8	11.04	6.58
Hexane	14.9	0	0	11.40	15.25
Propan-2-ol	15.8	6.1	16.4	11.82	7.33
Propan-1-ol	16	6.8	17.4	12.60	7.90
Ethanol	15.8	8.8	19.4	15.07	10.43
Acetonitrile	15.3	18	6.1	16.35	18.15
Methanol	14.7	12.3	22.3	19.77	15.44
Ethylene Glycol	17	11	26	20.93	15.70
Water	15.5	16	42.3	38.21	32.70

**Table 4:** Effect of solvent on ultrasonic extraction of CBD and Δ9-THC from cannabis powder.

Solvents	CBD			Δ9-THC		
	Mean Peak Area	SD	%RSD	Mean Peak Area	SD	%RSD
Dichloromethane	2185	64.60	2.96	25,793	596.41	2.31
Ethyl acetate	1628	17.82	1.09	19,262	143.51	0.75
Ethanol	1959	42.37	2.16	23,037	501.74	2.18

**Table 5:** Percentage of remaining standard CBD and Δ9-THC in ethanol solutions at a concentration of 100 µg/mL under ultrasonic extraction conditions.

Analytes	% Remaining				SD	%RSD
	1	2	3	Mean		
CBD	98.56	98.88	98.82	98.75	0.1694	0.17
Δ9-THC	99.96	100.11	100.27	100.11	0.1556	0.16

**Table 6:** Crude extract yield of Suk-Saiyasna commercial brand and in-house sample (*n* = 3).

Samples	Weight of Dried Powder (g)	Weight of Crude Extract (g) Mean ± SD	Percentage Yield Mean ± SD
Brand A	30	5.07 ± 0.15	16.89 ± 0.50
Brand B	30	4.71 ± 0.18	15.70 ± 0.60
Brand C	30	10.17 ± 0.38	33.89 ± 1.27
Brand D	30	3.86 ± 0.11	12.86 ± 0.37
Brand E	30	0.91 ± 0.14	3.12 ± 0.48
Brand F	30	1.73 ± 0.12	5.78 ± 0.40
In-house	30	4.92 ± 0.11	16.38 ± 0.37
Leaf	30	1.64 ± 0.08	5.47 ± 0.27

**Table 7:** Precursor and fragment ions including MS parameters used in the applied LC–MS/MS methods for Δ9-THC and CBD.

Analyte	*m*/*z*	Dwell Time (ms)	Q1 (V)	CE (V)	Q3 (V)
Δ9-THC	315.15 > 193.10	100	−13.7	−24.4	−18.1
	315.15 > 135.15	100	−13.7	−33.7	−10.8
CBD	315.20 > 193.10	100	−13.7	−21.1	−36.9
	315.20 > 123.05	100	−32.6	−21.5	−12.3
Tadalafil	390.10 > 268.10	100	−36.9	−16.6	−16.6
	390.10 > 135.10	100	−18.1	−21.0	−12.3

**Table 8:** Linearity, LOD, and LOQ for Δ9-THC and CBD.

Analyte	Linear Equation	R	LOD (ng/mL)	LOQ (ng/mL)
Δ9-THC	Y = 0.0376X + 0.0052	0.9999	0.9706	2.9412
	Y = 0.0378X + 0.0033	0.9999		
	Y = 0.0372X + 0.0233	0.9999		
CBD	Y = 0.0344X+ 0.0106	0.9999	0.3973	1.2039
	Y = 0.0344X + 0.0024	0.9999		
	Y = 0.0344X + 0.0056	0.9999		

**Table 9:** Accuracy and precision for Δ9-THC and CBD.

Analyte	Conc. (ng/mL)	Day	Mean Recovery (ng/mL)	% Recovery	SD	%RSD	%RSD Inter-Day
Δ9-THC	10.05	1	10.14	100.92	0.1352	1.33	1.09
		2	10.24	101.87	0.1947	1.90	
		3	10.42	103.72	0.2183	2.09	
	100.50	1	102.09	101.58	1.4503	1.42	2.06
		2	103.29	102.77	1.7348	1.69	
		3	99.48	98.98	1.0434	1.05	
	1005.00	1	991.52	99.10	13.2324	1.33	1.09
		2	1008.08	100.76	5.9643	0.59	
		3	997.88	99.74	7.3365	0.74	
CBD	10.05	1	10.06	100.15	0.1665	1.65	1.52
		2	10.00	99.51	0.1604	1.50	
		3	10.01	99.60	0.1920	1.92	
	100.50	1	100.00	99.51	1.9729	1.79	1.67
		2	99.92	99.42	1.8511	1.85	
		3	99.17	98.68	1.7667	1.78	
	1005.00	1	992.81	99.23	17.7839	1.79	1.51
		2	978.27	97.78	14.7668	1.58	
		3	978.76	97.83	14.8917	1.52	

**Table 10:** Extraction efficiency of dichloromethane on an extraction of Δ9-THC and CBD from Suk-Saiyasna remedy.

Number of Extractions	Extraction Efficiency of Dichloromethane on an Extraction of Δ9-THC (%)									
	In-House	Brand A	Brand B	Brand C	Brand D	Brand E	Brand F	Mean	SD	%RSD
1	84.67	93.53	88.15	80.30	83.26	80.45	83.28	84.81	4.68	2.72
2	99.93	99.93	99.91	100	99.91	99.92	99.94	99.93	0.03	0.03
3	100.00	100.00	100.00	100.00	100.00	100.00	100.00	100.00	0.00	0.00
Number of Extractions	Extraction Efficiency of Dichloromethane on an Extraction of CBD (%)									
	In-House	Brand A	Brand B	Brand C	Brand D	Brand E	Brand F	Mean	SD	%RSD
1	89.80	92.14	89.08	94.27	82.94	84.91	81.51	87.81	4.79	4.41
2	99.96	99.96	99.98	100	99.98	99.92	99.93	99.96	0.03	0.03
3	100.00	100.00	100.00	100.00	100.00	100.00	100.00	100.00	0.00	0.00

**Table 11:** The concentration of Δ9-THC in the crude extracts of commercial and in-house Suk-Saiyasna herbal remedies at 10 mg/mL.

Sample	Crude Extract (g) Mean ± SD	Δ9-THC (ng/mL) Mean ± SD	Δ9-THC (mg) in 30 g Herbal Remedy Mean ± SD	Δ9-THC (%) in 30 g Herbal Remedy	Δ9-THC (%) in Cannabis Leaves 4.61 g
Brand A	5.07 ± 0.15	12,941.36 ± 387.88	6.56 ± 0.20	0.02187	0.14218
Brand B	4.71 ± 0.18	5904.82 ± 247.97	2.78 ± 0.12	0.00927	0.06028
Brand C	10.17 ± 0.38	108.54 ± 10.71	0.11 ± 0.01	0.00036	0.00231
Brand D	3.86 ± 0.11	3260.20 ± 129.07	1.22 ± 0.05	0.00407	0.02649
Brand E	0.91 ± 0.14	6295.47 ± 441.04	0.59 ± 0.04	0.00195	0.01271
Brand F	1.73 ± 0.12	10,462.97 ± 698.66	1.82 ± 0.12	0.00607	0.03947
In-house	4.92 ± 0.11	8521.71 ± 216.89	4.19 ± 0.01	0.01397	0.09084
Leaf	1.64 ± 0.08	27,181.19 ± 732.50			0.09661

**Table 12:** The concentration of CBD in the crude extracts of commercial and in-house Suk-Saiyasna herbal remedies at 10 mg/mL.

Sample	Crude Extract (g) Mean ± SD	CBD (ng/mL) Mean ± SD	CBD (mg) in 30 g Herbal Remedy Mean ± SD	CBD (%) in 30 g Herbal Remedy	CBD (%) in Cannabis Leaves 4.61 g
Brand A	5.07 ± 0.15	1245.50 ± 108.72	631.34 ± 55.11	0.00210	0.01368
Brand B	4.71 ± 0.18	1509.68 ± 108.10	711.06 ± 50.92	0.00237	0.01541
Brand C	10.17 ± 0.38	0.87 ± 0.07	0.86 ± 0.07	0.00000	0.00002
Brand D	3.86 ± 0.11	1619.38 ± 67.76	607.13 ± 25.20	0.00202	0.01316
Brand E	0.91 ± 0.14	167.09 ± 8.13	15.56 ± 0.72	0.00005	0.00034
Brand F	1.73 ± 0.12	1084.84 ± 59.38	188.82 ± 10.43	0.00063	0.00409
In-house	4.92 ± 0.11	19.97 ± 0.60	9.82 ± 0.29	0.00003	0.00021
Leaf	1.64 ± 0.08	67.16 ± 0.72			0.00024

## Data Availability

Data are contained within the article.
